# Supplementary material for: Circadian Intervention Improves Parkinson’s Disease and May Slow Disease Progression: A Ten Year Retrospective Study
Source: Brain Sci. 2024 Nov 30;14(12):1218. doi: 10.3390/brainsci14121218 (PMC11726833; doi:10.3390/brainsci14121218)
Supplement: Supplementary file 1 [file brainsci-14-01218-s001.zip › brainsci-3316836-supplementary.pdf]

Supplementary Material

Fig. S1a: Elbow to Fist Latency: 2012

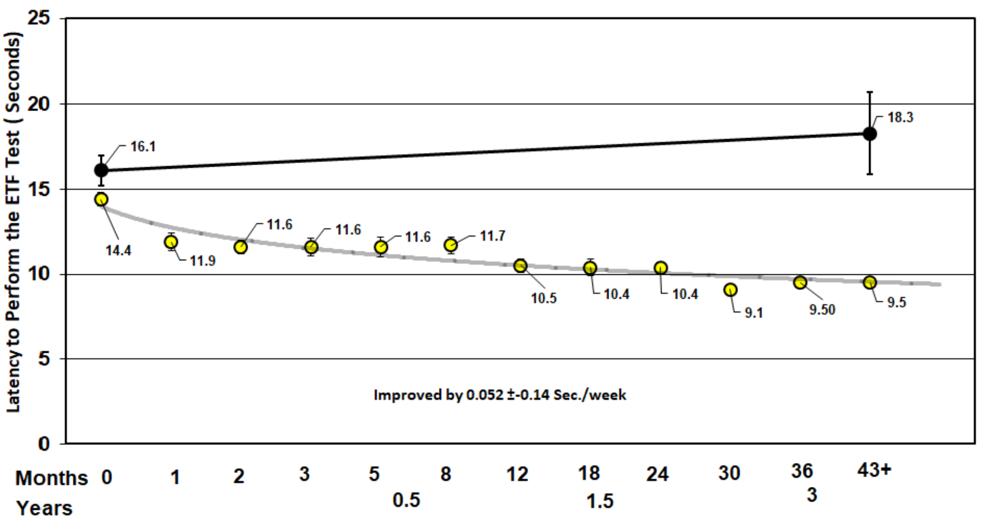

Fig. S1b: Floor to Knee Latency: 2012

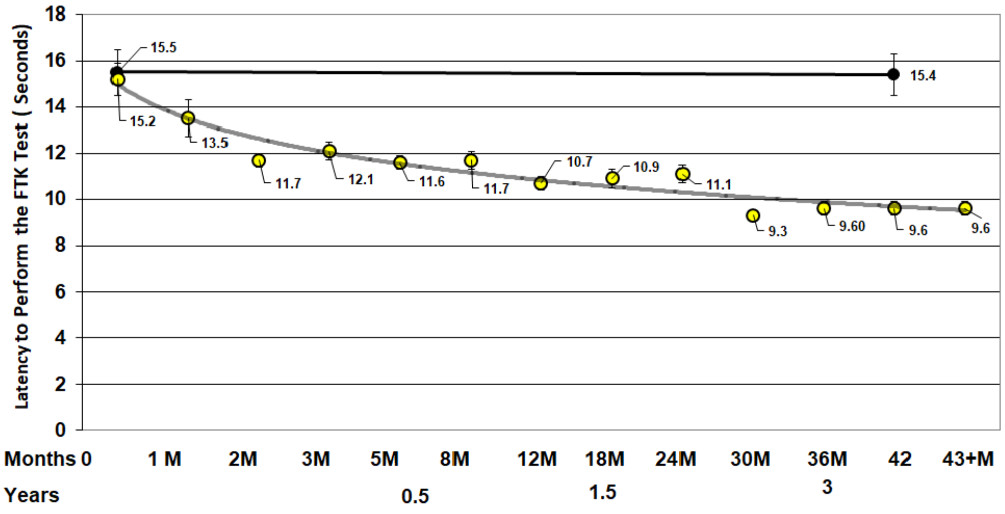

Figure S1: The effect of phototherapy over a five year (plus) period on the timed motor tests (Measured in seconds). Fig. S1a shows the results of the elbow to fist (ETF) test at each point of observation as published previously [7]. The group that improved on average test performance (10 revolutions) were designated as being fully compliant (yellow circles). Those that showed an average performance that was longer showing deterioration compared to their pre-treatment measurement were designated non-compliant (black circles) as they exited the program early (n=28). The first data point on the left represents the pre-treatment value for both groups. Fig. S1b depicts the results of the floor to knee (FTK) test at each point for those that exited the program early. As per Fig. S1a, the first data point represented on the left for each group represents the pre-treatment value for the responsive and the non-responsive groups and the T-bars represent the standard error of the mean. This data illustrates a similar response reported in the novel data of the present study and was reproduced with permission. (From Willis et al, 2012 [7] reproduced with Permission from Walter de Gruyter Publishers).

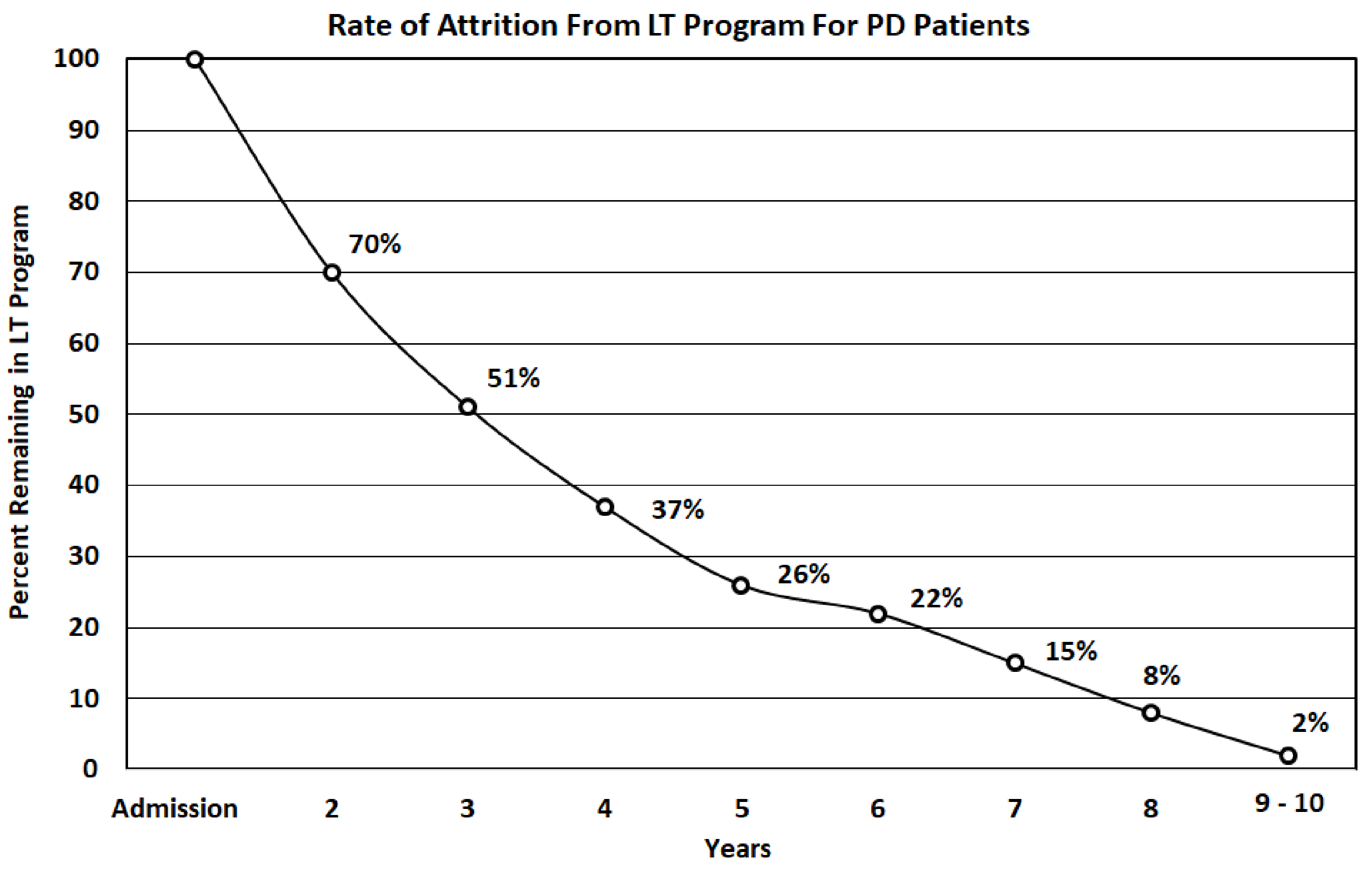

Figure S2: The yearly rate of drop out in patient number from the light treatment program over a 10 year period. The rate of drop out is expressed as percent original total number remaining for each year after admission.
